# Molecular epidemiology of *Dientamoeba fragilis* coinfections with *Blastocystis* sp. and *Giardia duodenalis* in symptomatic patients from Valencia, Spain

**DOI:** 10.1007/s10096-026-05586-2

**Published:** 2026-07-09

**Authors:** Marta García-Hita, Jorge Ferriz, Susana Cifre, Gabriela Tapia-Veloz, Araceli Molina, David Carmena, Maria Trelis

**Affiliations:** 1 Department of Clinical Microbiology, University Hospital Los Arcos del Mar Menor, Murcia, Spain; 2 Severe Infection Research Group, Medical Research Institute La Fe, Valencia, Spain; 3 Department of Clinical Analysis Laboratory, Sierra de Segura High Resolution Hospital, Jaen, Spain; 4 Area of Parasitology, Department of Pharmacy and Pharmaceutical Technology and Parasitology, University of Valencia, Valencia, Spain; 5 Department of Clinical Microbiology, Arnau de Vilanova Hospital, Valencia, Spain; 6 Parasitology Reference and Research Laboratory, Spanish National Centre for Microbiology, Health Institute Carlos III, Majadahonda, Spain; 7 Center for Biomedical Research Network (CIBER) in Infectious Diseases, Health Institute Carlos III, Madrid, Spain; 8 Joint Research Unit on Endocrinology, Nutrition and Clinical Dietetics, Health Research Institute La Fe, Valencia, Spain

**Keywords:** *Dientamoeba fragilis*, Intestinal protists, Molecular characterization, Genotype

## Abstract

**Purpose:**

*Dientamoeba fragilis* is a cosmopolitan intestinal protist with an insufficiently understood life cycle and pathogenicity. Diagnosis by light microscopy is challenging, leading to significant underdiagnosis. Two genotypes (1 and 2) have been described, with genotype 1 being the most frequently detected worldwide. As no molecularly characterized isolates from Spain have been reported, we performed a molecular characterization and evaluated sociodemographic factors of *D. fragilis* in patients coinfected with *Blastocystis* sp. and/or *Giardia duodenalis*.

**Methods:**

Using a multiplex qPCR assay (Allplex™ GI-Parasite Assay, Seegene), we analyzed 354 stool samples previously identified as positive for *Blastocystis* sp. (*n* = 276), *G. duodenalis* (*n* = 63), or both (*n* = 15) by light microscopy. Eligible positive samples were selected for conventional PCR targeting the *SSU* rRNA gene and subsequent Sanger sequencing.

**Results:**

*D. fragilis* was detected in 34.5% of samples by qPCR; 32.3% of *Blastocystis*-positive, 44.9% of *Giardia*-positive and 46.7% of coinfected samples. We successfully sequenced 22 isolates, all corresponding to genotype 1, identifying two distinct intra-genotypic sequence variants. Younger age was positively associated with *D. fragilis*, with individuals coinfected with *Blastocystis* sp. being markedly younger than those monoinfected. Patients with *G. duodenalis* and *D. fragilis* were mainly under 15 years of age.

**Conclusion:**

This study provides the first genotyping of *D. fragilis* in Spain, demonstrating the circulation of two genotype 1 variants. Findings highlight the underdiagnosis of *D. fragilis* and its high prevalence in young patients with other fecal-orally transmitted protists. Evaluating coinfections is crucial to understanding parasite relationships and should be considered for clinical management, especially when symptoms persist.

## Introduction

*Dientamoeba fragilis* is a single-celled protozoan commonly found in the human gastrointestinal tract that was first described over a century ago [1]. It was traditionally classified as an amoeba due to the absence of flagella. However, advances in electron microscopy and molecular phylogenetic studies have led to its reclassification within the order Trichomonadida [2, 3]. Several aspects of the biology, epidemiology, and public health relevance of *D. fragilis* remain poorly understood. Key unresolved issues include the significance of its different morphological forms, its actual prevalence, modes of transmission, pathogenic potential, and the treatment of choice, all of which continue to be source of controversy [4].

*Dientamoeba fragilis* has a cosmopolitan distribution and has been widely reported worldwide in both developed and developing countries, but its true prevalence remains unknown and varies significantly across studies, ranging from 0.2 to 82.9% [5–7]. Furthermore, this prevalence is likely underestimated due to variations in study design, populations, and diagnostic techniques. Higher prevalence rates have been observed in developed countries, probably attributable to increased use of molecular detection methods.

The pathogenic potential of *D. fragilis* remains a subject of ongoing controversy. Several studies, including clinical reports and descriptions of symptomatic cohorts, have observed an association between the presence of this protozoan and gastrointestinal symptoms—predominantly diarrhea, abdominal pain, and nausea—as well as irritable bowel syndrome (IBS) and peripheral eosinophilia [8–10]. However, many of these findings are based on symptomatic cases lacking appropriate control groups, and conversely, other structured case-control studies have reported a high or even higher prevalence of the microorganism among asymptomatic individuals [11, 12]. This lack of consensus has led to *D. fragilis* being frequently omitted from routine testing in clinical laboratories, often classifying it as an emerging but ‘neglected parasite’ [13].

No clear associations have been found between the presence of *D. fragilis* and specific sociodemographic factors such as sex or hygiene practices, including handwashing and consumption of tap water [14, 15]. The relationship with age has not been clearly established either. Some studies have shown higher detection rates of *D. fragilis* in young patients (0–24 years) [15–17], with a bimodal age distribution peaking in children and adults of parental age [6, 18]; however, other studies have found *D. fragilis* to be more frequent in adults [19].

The life cycle and transmission mechanism of *D. fragilis* are also not fully understood. Historically, only the trophozoite stage was known. This stage inhabits the caecum and ascending colon, but its fragility outside the host makes direct human-to-human transmission difficult to explain [20]. However, the existence of precyst and cyst stages has been proposed by some authors, considering them as the infectious forms responsible for fecal-oral transmission [21, 22]; this could explain the high prevalence of *D. fragilis* in contacts of patients harboring the protist (up to 50%) [18, 23]. Although it has been observed that some animals, such as non-human primates, rodents, pigs, cattle and birds, can serve as natural hosts of *D. fragilis*, the possibility of zoonotic transmission remains unclear [24–27]. Nevertheless, the cystic forms predominantly found in the feces of mice could be a significant factor in zoonotic transmission [20]. Other authors postulate the hypothesis that the eggs of the helminth *Enterobius vermicularis* could play an important role as a vector for the transmission of *D. fragilis*. The fact that *Histomonas meleagridis*, the phylogenetically closest organism to *D. fragilis*, has a helminth vector would support this theory [28, 29].

Although the pathogenic potential of *D. fragilis* is still debated, it’s often treated with a single-drug regimen that differs throughout the world [30]. Paromomycin is usually the empirical treatment of choice, with metronidazole, ornidazole, and doxycycline as alternatives [30–32]. In several observational cohorts, clinical improvement or symptom resolution has been reported following successful parasite eradication [33, 34]. However, data from randomized placebo-controlled trials indicate that the clearance of this protozoan does not significantly influence the long-term health status or clinical outcomes of patients with chronic digestive disorders [35].

Given the fragility in environmental conditions of *D. fragilis* trophozoites, direct identification in fresh stool samples without permanent staining, such as trichrome or modified iron hematoxylin, is discouraged. These light microscopy techniques show low diagnostic performance and are being replaced by molecular methods, such as real-time PCR (qPCR) or multiplex tandem PCR (MT-PCR), which provide higher sensitivity than conventional methods [36]. The adoption of these molecular methods has resulted in improved detection rates among patients with gastrointestinal symptoms and has become the current gold standard for *D. fragilis* [37]. Furthermore, molecular characterization based on amplification of a partial fragment of the small subunit ribosomal ribonucleic acid (*SSU* rRNA) gene of *D. fragilis* allow to distinguish two genotypes, genotype 1 and genotype 2 (also referred to as Bi/PA strain). These genotypes exhibit low genetic diversity, with genotype 1 being the most frequently reported in human [38]. Available evidence indicates that analyses of intragenic variation in constitutive genes, such as actin and elongation factor 1 alpha (EF-1α), have limited epidemiological and clinical utility in studies of *D. fragilis* in humans [13]. To date, no differences in pathogenicity between genotypes have been demonstrated [12, 13, 39].

In recent years, several commercial PCR kits have been developed for the simultaneous detection of gastrointestinal protists, such as *Blastocystis* sp. and *Giardia duodenalis*, along with *D. fragilis*, demonstrating good sensitivity and specificity [40, 41]. *Blastocystis* sp., *G. duodenalis*, and *D. fragilis* are the most frequently reported microeukaryotes in fecal samples from patients with gastrointestinal symptoms [13, 42, 43]. Due to possible shared environmental risk factors, these protists frequently coexist in human hosts [32, 43, 44]. While the pathogenic role of *G. duodenalis* is well established [45], the clinical significance of *Blastocystis* sp. and *D. fragilis* remains a subject of debate [46].

The main objective of the present study is to improve the available knowledge about *D. fragilis*, specifically in cases of coinfection with *Blastocystis* sp. and *G. duodenalis*, as well as provide the first molecular characterization of this protist carried out in Spain.

## Materials and methods

This observational, descriptive, cross-sectional molecular epidemiological study was conducted in 2025 from stored DNA of stool samples (*n* = 354), collected from 2017 to 2019, from patients with suspected parasitosis who tested positive for *Blastocystis* sp. (*n* = 276), *G. duodenalis* (*n* = 63) or both (*n* = 15) by light microscopy in a wet mount. In two of these samples (0.6%), one containing *Blastocystis* sp. and another with *G. duodenalis*, *D. fragilis* was also observed microscopically. Stool sample collection was performed using the Kit REAL MiniSystem Easy Pick System with Total-Fix^®^ (Durviz, Valencia, Spain).

At the time of receiving the fecal samples, they were concentrated and filtered and the sediment obtained was intended for coproparasitological examination by microscopy. Genomic DNA of positive stool samples for microscopy was isolated by using the QIAamp^®^ Fast DNA Stool Mini Kit (QIAGEN, Hilden, Germany) according to the manufacturer’s recommendations. The purified DNA was eluted in 100 µL of PCR-grade water and stored at 4 °C until further molecular analysis.

### Multiplex real-time PCR assay

Multiplex real-time PCR assayStool DNA samples were screened using the Allplex™ GI-Parasite Assay (Seegene, Seoul, South Korea). This technique, based on MuDT™ (Multiple Detection Temperatures) technology, allows the simultaneous detection of several parasites (*Blastocystis* sp., *Cryptosporidium* spp., *Cyclospora cayetanensis*, *Dientamoeba fragilis*, *Entamoeba histolytica* and *Giardia duodenalis*) through real-time PCR (qPCR) [41]. PCR reaction mixtures were prepared to a final volume of 25 µL by adding 5 µL of 5x Primers MoM, 5 µL of 5x Anyplex PCR Master Mix (EM2), 8 µL of RNases-free water, 2 µL of internal control and 5 µL of DNA template. Positive (provided by the manufacturers) and negative (ultrapure water) controls were included in all reactions. The amplification cycle was: 20 min at 50 °C, 15 min denaturation hold at 95 °C; 44 cycles of 10 s at 95 °C, 1 min at 60 °C and 30 s at 72 °C. Subsequently, the results were analyzed using the Seegene Viewer^®^ software for the visualization of reaction curves and determination of cycle threshold (C_t_) values, establishing a cut-off point of C_t_ ≤ 38.

### Molecular characterization

To ensure sufficient target DNA abundance and optimize yield for downstream sequencing, samples with a C_t_ ≤ 29 for *D. fragilis* were selected for genotyping using direct conventional PCR (cPCR) targeting the *SSU* rRNA gene of the protist and subsequent Sanger sequencing of the generated amplicons. To amplify a PCR product of ~ 850 bp, we employed the specific primers DF400 (5′–TATCGGAGGTGGTAATGACC–3′) and DF1250 (5′–CATCTTCCTCCTGCTTAGACG–3′) (Invitrogen, California, USA) [47]. PCR reaction mixtures were prepared in a final volume of 25 µL by adding 2 µL of DNA template, 5 µL 5x MyTAQ™ Reaction Buffer containing 5 mM dNTPs and 15 mM MgCl_2_ (Bioline GmbH, Luckenwalde, Germany), 0.5 µL of 5 U/µl MyTAQ™ DNA polymerase (2.5 U) (Bioline GmbH), 1.25 µL of each primer, 0.5 µL of 10 mM dNTPs mix (Invitrogen) and 14.5 µL of ultrapure water. Amplification conditions consisted of one step of 94 °C for 3 min, followed by 30 cycles of 1 min at 94 °C, 1.5 min at 57 °C and 2 min at 72 °C, with an additional 7 min for final extension at 72 °C [47]. We used laboratory-confirmed positive and negative DNA samples as controls. PCR amplicons were visualized on 2.0% Certified Genetic Quality Tested (GQT) DNA Grade Agarose (Bio-Rad, California, USA) stained with SafeView (NBS Biologicals, Huntingdon, UK). The GeneRuler 100 bp DNA Ladder (Thermo Fisher Scientific, Massachusetts, USA) was used for sizing the obtained amplicons.

Raw sequencing data obtained by Sanger, in both forward and reverse directions, were viewed using Chromas 2.6.6 (Technelysium Pty Ltd., South Brisbane, Australia). *SSU* rRNA sequences obtained were subsequently checked for local alignment in the NCBI GenBank database (https://blast.ncbi.nlm.nih.gov/Blast.cgi) using the Nucleotide BLAST tool [48]. To generate DNA consensus sequences and detect the presence of ambiguous positions (double peak), chromatograms were inspected using Chromas 2.6.6 and MEGA-X v11.0.13 software (Molecular Evolutionary Genetics Analysis), aligning with reference sequences AY730405.1 (genotype 1) and U37461.1 (genotype 2).

### Phylogenetic analysis

A phylogenetic analysis was conducted to infer a tree based on partial-length *SSU* rRNA gene sequences from different isolates obtained from GenBank (Table 1). This analysis, based on the Neighbor-Joining method, was performed using the MEGA-X 11 v11.0.13 software. The evolutionary distances were computed using the Tamura 3-parameter method with 1,000 bootstrap iterations. Rate variation among sites was modelled with a gamma distribution (shape parameter = 2).

**Table 1:** Summary of accession numbers (GenBank) used in the phylogenetic tree

Accession number	Host	Country	Sequence length (bp)†	References
Genotype 1				
AY730405*	Human	Australia	1661	[47]
FJ649228	Human	Australia	792	[37]
OP375681	Human	Czech Republic	859	[25]
OP375685	Human	Czech Republic	871	[25]
OQ345680	Human	Italy	748	[49]
OR921936	Human	Italy	748	[50]
JQ677149	Human	Italy	1085	[24]
JQ677148	Human	United Kingdom	1501	[24]
AB692772	Human	Iran	856	[51]
MN914083	Human	Germany	1621	[52]
OM250406	Human	Turkey	788	[53]
OP375700	Rabbit	Czech Republic	845	[25]
ON242172	Cattle	Turkey	810	[26]
Genotype 2				
U37461*	Human	USA	1676	[2]
Other organisms				
AF293056 (*Histomonas meleagridis*)	-	Turkey	1602	[54]
U17510 (*Trichomonas vaginalis*)	-	-	1574	[55]
U37711 (*Trichomonas tenax*)	-	-	1580	Unpublished
AF124609 (*Pentatrichomonas* spp.)	-	France	1504	[56]
EU647889 (*Parahistomonas wenrichi*)	Bird	France	1625	[57]

*Reference sequences

**†**Sequence length refers to the total obtained sequencing yield; all isolates were subsequently aligned and trimmed to a common 698-bp region for phylogenetic analysis

We included 13 sequences of genotype 1 (11 of human origin and 2 of animal origin) and one sequence of genotype 2 (human origin). Additionally, sequences from other species within the order Trichomonadida (*Histomonas meleagridis*, *Parahistomonas wenrichi*,* Pentatrichomonas* sp., *Trichomonas tenax*, *Trichomonas vaginalis*) were included in the analysis to explore the evolutionary relationships between these taxa and *D. fragilis* sequences (Table 1).

### Statistical analysis

Graphs and statistical analyses were conducted using SPSS v25.0 and R Studio software. The data didn´t follow a normal distribution and a descriptive and comparative analysis of the variables of interest was performed using non-parametric tests: Mann–Whitney U and Chi-square tests (χ^2^). P-values < 0.05 were considered statistically significant.

### Ethics statement

This study was approved by the Biomedical Research Ethics Committee of University and Polytechnic Hospital La Fe on 2025 (Project identification code: 2025-0418-1), respecting the fundamental principles of the Declaration of Helsinki, of the Council of Europe Convention in relation to Human Rights, and Biomedicine of the UNESCO Declaration.

## Results

By multiplex qPCR, *D. fragilis* was detected in 34.5% (122/354; 95% CI: 29.5–39.7) of the analyzed samples. The same assay confirmed the presence of *Blastocystis* sp. in 291 samples and *G. duodenalis* in 78 samples. *Dientamoeba fragilis* was identified in 32.3% (94/291) of the *Blastocystis*-positive samples, in 44.9% (35/78) of the *Giardia*-positive samples, and in 46.7% (7/15) of samples coinfected with both *Blastocystis* sp. and *G. duodenalis*. All samples tested negative for *Cryptosporidium* spp., *C. cayetanensis*, or *E. histolytica*.

The C_t_ values of *Blastocystis*- and *Giardia*-positive samples obtained by qPCR were compared to infer their relative parasite loads of both protists in case of coinfection with *D. fragilis*. The C_t_ value of *Blastocystis*-positive samples was significantly lower in patients coinfected with *D. fragilis* (28.3 ± 5.2) than in patients without coinfection (29.8 ± 6.2) (*p* < 0.05), suggesting a relatively higher *Blastocystis* load in coinfected individuals. Moreover, a moderate positive correlation was observed between the C_t_ values of *Dientamoeba*-positive samples and those of *Blastocystis*-positive samples (*r* = 0.516; *p* < 0.001). In contrast, no significant differences in C_t_ values (*p* = 0.888) or correlations (*r* = 0.118, *p* = 0.501) were observed between samples positive for *G. duodenalis* and *D. fragilis*.

### Sociodemographic analysis

Overall, 51.9% (151/291) of *Blastocystis*-positive samples were obtained from female patients, with this percentage being slightly higher (56.4%, 53/94) among cases coinfected with *D. fragilis*. However, no statistically significant association with sex was observed among patients coinfected with *Blastocystis* sp. and *D. fragilis* (χ^2^ = 1.123; *p* = 0.289) (Table 2).

**Table 2:** Sociodemographic factors associated with *Blastocystis* sp. and *D. fragilis* coinfection cases. Statistically significant values are bolded

	*Blastocystis sp.* *n*, (%)	*Blastocystis sp. alone* *n*, (%)	*Blastocystis sp.* + *D. fragilis* *n*, (%)	*p*-value
Total	291 (100)	197 (67.7)	94 (32.3)	-
Sex				
Female	151 (51.9)	98 (64.9)	53 (35.1)	0.289
Male	140 (48.1)	99 (70.7)	41 (29.3)	
Age				
Children (≤ 15 yrs.)	91 (31.3)	43 (47.3)	48 (52.7)	**< 0.001**
Adults (> 15 yrs.)	200 (68.7)	154 (77.0)	46 (23.0)	
Season				
Spring	73 (25.1)	45 (61.6)	28 (38.4)	0.06
Summer	61 (21.0)	39 (63.9)	22 (36.1)	
Autumn	85 (29.2)	55 (64.7)	30 (35.3)	
Winter	72 (24.7)	58 (80.1)	14 (19.9)	

The mean age of patients positive for *Blastocystis* sp. was 37.9 years (± 24.9). Patients colonized solely by *Blastocystis* sp. were significantly older (43.1 ± 24.4 yrs.) than those coinfected with *D. fragilis* (27.1 ± 22.5 yrs.; *p* < 0.001). When patients were stratified by age (≤ 15 years for children and > 15 years for young adults and adults), a significantly higher rate of coinfection with *D. fragilis* was observed among younger individuals positive for *Blastocystis* sp. (52.9%, 48/91) compared with adults (23.0%, 46/200) (χ²= 25.308; *p* < 0.001). In contrast, no significant differences were observed for coinfections with *G. duodenalis* (χ²= 0.533; *p* = 0.465). No statistically significant association was found between *Blastocystis* sp. and *D. fragilis* coinfection and the season of sample collection (χ^2^ = 7.410; *p* = 0.06).

Among patients infected with *G. duodenalis*, women accounted for 42.3% (33/78), of whom 45.7% (16/33) were coinfected with *D. fragilis* (Table 3).

**Table 3:** Sociodemographic factors associated with *G. duodenalis* and *D. fragilis* coinfection

	*G. duodenalis* *n*, (%)	*G. duodenalis alone* *n*, (%)	*G. duodenalis* + *D. fragilis* *n*, (%)	*p*-value
Total	78	43 (55.1)	35 (44.9)	-
Sex				
Female	33 (42.3)	17 (51.5)	16 (45.7)	0.583
Male	45 (57.7)	26 (57.8)	19 (42.2)	
Age				
Children (≤ 15 yrs.)	57 (73.1)	30 (52.6)	27 (47.4)	0.465
Adults (> 15 yrs.)	21 (26.9)	13 (41.9)	8 (38.1)	
Season				
Spring	8 (10.3)	3 (37.5)	5 (62.5)	0.540
Summer	26 (33.3)	13 (50.0)	13 (50.0)	
Autumn	32 (41.0)	19 (59.4)	13 (40.6)	
Winter	12 (15.4)	8 (66.7)	4 (33.3)	

No significant sex-based differences were observed in coinfected patients (χ²= 0.303; *p* = 0.583). The overall mean age of *Giardia*-positive patients was low (15.3 ± 18.8 yrs.) with only 26.9% (21/78) being adults. No significant age differences were found between patients with coinfection (14.2 ± 17.6 yrs.) and those without (16.1 ± 19.9 yrs., *p* = 0.353). Furthermore, there was no significant association between *G. duodenalis* and *D. fragilis* coinfection and the season of sample collection (χ^2^ = 2.161; *p* = 0.540).

### Molecular characterization

Overall, 28.7% (35/122) of the *D. fragilis*-positive isolates detected by qPCR yielded amplifications with C_t_ values ≤ 29. Among these, the success rate of *SSU*-PCR was 62.9% (22/35), corresponding to 19 samples coinfected with *Blastocystis* sp. and 3 samples coinfected with *G. duodenalis*. All sequencing data covered a 698-bp region (positions 481 to 1178) of the reference sequence for genotype 1 (GenBank Acc. No. AY730405).

The *SSU* rRNA gene sequences obtained in this study have been deposited in GenBank under accession numbers PX606388-PX606409. Compared to the reference sequence of genotype 1 (AY730405), 21 sequences (PX606388-PX606408) showed 99.7% identity, with a single nucleotide deletion at position 629 (G) and a substitution of C→T at position 696. In comparison, the reference sequence of genotype 2 (U37461) showed 94.9% identity with characteristic single nucleotide polymorphisms confirming that all isolates in this survey belong to genotype 1. BLAST analysis revealed that our sequences were 100% identical to multiple isolates from different countries, such as Australia (FJ649228), Czech Republic (OP375687), Iran (AB692772), Italy (OQ345680), Turkey (MN560149) and United Kingdom (JQ677147) with humans as the hosts in all cases except for ON24217 from Turkey, which was isolated from cattle.

The remaining isolate (PX606409) differed from the others (PX606388-PX606408) by an insertion (T) at position 923, a feature also seen in two isolates from the Czech Republic (OP375694 and OP375698). Despite this difference, it still showed a high level of identity with the genotype 1 reference sequence (99.6%) compared to the genotype 2 reference sequence (94.9%), and was therefore classified as genotype 1.

### Phylogenetic analysis

**Fig. 1:**
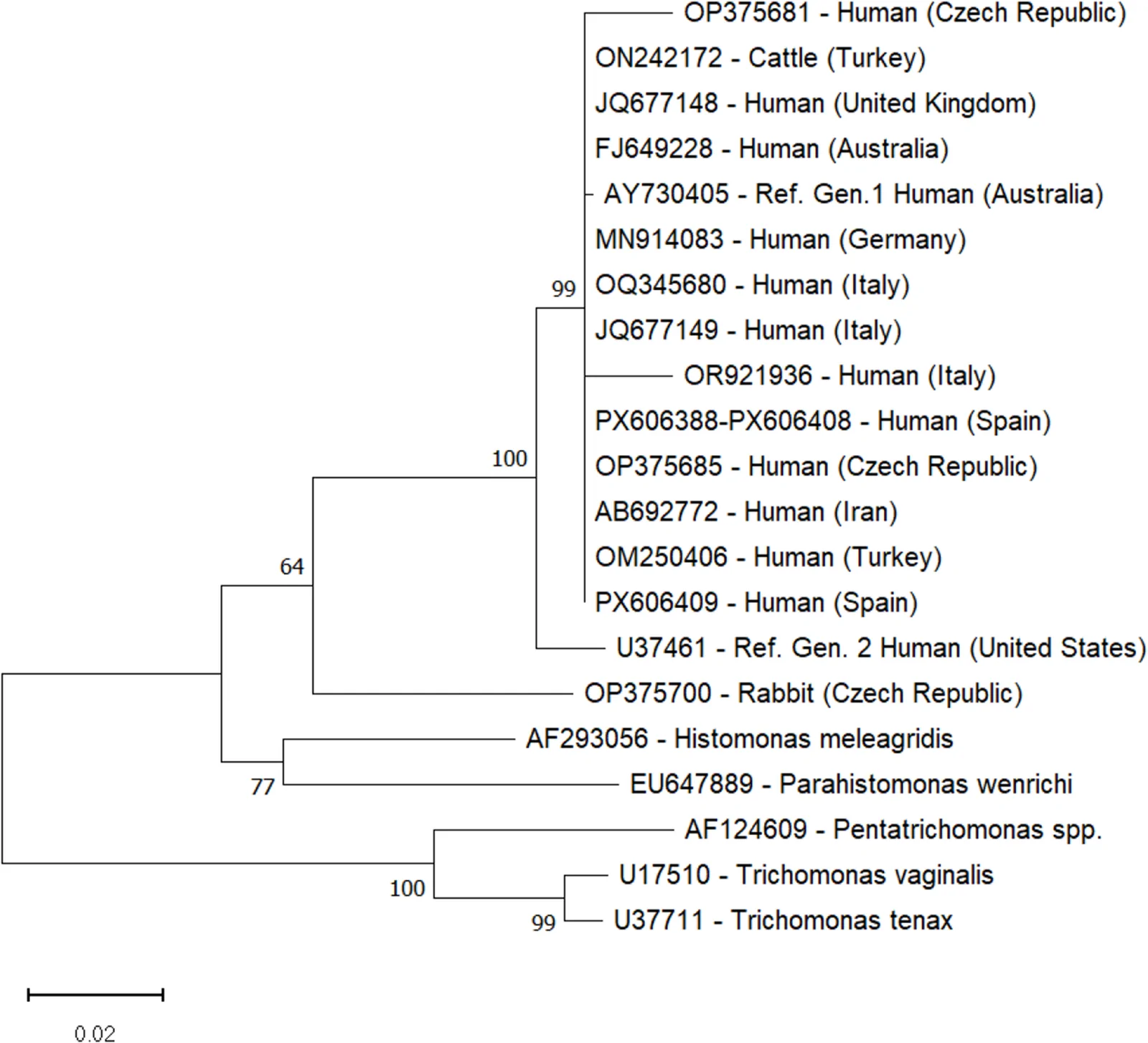
Evolutionary relationships among *D. fragilis SSU* rRNA isolates, based on a Neighbor-Joining analysis of the nucleotide sequence spanning a 698-bp region of the reference sequence for genotype 1 (positions 481 to 1178 of GenBank Acc. No. AY730405). The percentage of replicate trees in which the related taxa grouped together during the bootstrap test (1,000 iterations) is shown next to the branches. Bootstrap values below 50% were omitted

The phylogenetic tree shows a distinct clade encompassing all *D. fragilis* isolates, with the exception of a rabbit-derived isolate (OP375700) (Fig. 1). Within this clade, a large subclade corresponded to genotype 1, while a smaller subclade contained the single genotype 2 isolate.

As expected, all sequences generated in this survey were unambiguously assigned to the genotype 1 subclade. Other related species included in the analysis, all belonging to the order Trichomonadida, resolved as separate, well-supported lineages.

## Discussion

Our study confirms that *D. fragilis* is frequent in patients with suspected gastrointestinal illnesses in coinfection with *Blastocystis* sp. (32.3%) and *Giardia duodenalis* (44.9%) and it can remain underdiagnosed in routine test at the hospitals that don´t include molecular methods, such as real-time PCR (qPCR) or multiplex tandem PCR (MT-PCR). In our study, the sensitivity of light microscopy was merely 1.6% (2/122) relative to qPCR detection. When using microscopy with permanent stains, correct and timely sample preparation is essential, as *D. fragilis* is known to lose viability and degrade quickly after being passed from the host. Several studies conducted in Spain have yielded similar results, where the use of PCR significantly increases the detection rate of *D. fragilis* [58, 59], which even emerges as the predominant protozoa [59]. Additional research in our country has described the clinical and epidemiological characteristics of *D. fragilis*, with prevalence rates ranging from 1.6% to 50.5% [10, 33, 42, 59].

Regarding coinfection, studies assessing the presence of *D. fragilis* in patients with *Blastocystis* sp. have reported prevalence rates ranging from 16.7% to 34.8% [16, 60, 61]. In contrast, no studies have evaluated the presence of *D. fragilis* in cohorts of patients with *G. duodenalis*. In studies in patients with *D. fragilis*, coinfection rates were observed to range from 17.8% to 47.4% with *Blastocystis* sp [14, 25, 32, 33, 44, 62]. and from 2.4% to 16.6% with *G. duodenalis* [10, 33, 62]. Therefore, the results of our study highlight a higher rate of coinfection by these protists in Spain, particularly between *D. fragilis* and *G. duodenalis* reaching 44.9%.

Several studies have explored these associations, particularly between *Blastocystis* sp. and *D. fragilis*, which may be capable of long-term colonization [63]. Both protists may share common transmission patterns and a possible interaction between them [61]. One study also suggests an increased likelihood of carrying *D. fragilis* in patients with *Blastocystis* sp [9]. While a statistically significant association between the two protists was found [16], no association was detected between a particular *Blastocystis* sp. subtype and *D. fragilis* in coinfection [16, 60]. Our findings are consistent with this association, as we observed a significantly higher relative *Blastocystis* load in patients coinfected with *D. fragilis*. Additionally, the moderate positive correlation between their C_t_ values (*r* = 0.516; *p* < 0.001) might reflect shared acquisition routes, common risk factors or a potential underlying biological interaction that warrants further experimental investigation.

Regarding *G. duodenalis*, the higher frequency of coinfection could be attributed to the fact that both protists are more common at certain ages (children and adults of parental age), probably due to more susceptibility to enteric infections in children as a result of poor hygiene and the close contact with their parents [7]. Unfortunately, few studies analyze other aspects of the interactions between these protists.

On the other hand, *Blastocystis* sp. has been reported to be more prevalent in adults and elderly individuals compared to children, likely due to repeated exposure to the protist throughout life and its role as a permanent member of the intestinal microbiota of the host [64, 65]. As a result, significant differences are observed in our data examining age and the presence or absence of coinfection of *Blastocystis* sp. and *D. fragilis*, suggesting that *D. fragilis* primarily affects children (≤ 15 years).

Although no differences were observed regarding the season of the year and the detection rate of *D. fragilis*, another study suggested a seasonal variation in *D. fragilis* carriage, finding increased prevalence in autumn [6]. However, these findings were not replicated in a subsequent study [42].

With the increasing accessibility of more modern DNA-based technologies, and with rapid extraction and purification of stool DNA from fresh samples, higher *D. fragilis* detection rates can be expected, as well as a more precise phenotypic and genotypic characterization, which will allow the elucidation of the underlying molecular mechanisms of its pathogenesis and a better understanding of its relationship with gastrointestinal diseases.

To our knowledge this is the first genotypic characterization of *D. fragilis* isolates in patients from Spain. Selecting isolates with values C_t_ ≤ 29 in the qPCR assay allowed us to achieve a high amplification yield, resulting in a 62.9% (22/35) success rate for the *SSU*-PCR and subsequent sequencing. Furthermore, a low genetic diversity was observed among these sequences, with all isolates classified as genotype 1, identifying two distinct intra-genotypic sequence variants, similar to that found in other countries such as Czech Republic [25], Italy [24, 49], Australia [37], Iran [51] or Turkey [53], all of them with almost complete genetic homogeneity, consistent with the clonal nature of the species. These results align with other studies demonstrating that *D. fragilis* shows remarkably little variation in its *SSU* rRNA gene [38, 39, 66] with only two genotypes that recently diverged. Moreover, the sequence divergence between the *SSU* rRNA genes of genotype 1 (AY730405) and genotype 2, strain Bi/PA (U37461), is approximately 4.0% within the DNA fragment examined using the primers employed in this study [47]. To date, genotype 2 data is scarce, and it has not been possible to establish a clear and consistent relationship between the genotype of *D. fragilis* and coinfection with other parasites or its ability to cause disease [39]. The absence of genotype 2 in our study aligns with global epidemiology; this genotype is only exceptionally found in human and animal surveys worldwide, where genotype 1 clearly predominates [7, 13]. Therefore, although the number of sequenced samples is limited, given the high genetic homogeneity and clonal nature globally described for *D. fragilis*, it is highly probable that genotype 1 remains dominant across other isolates in our region.

To perform the molecular characterization of *D. fragilis*, selecting patients already harboring *Blastocystis* sp. or *G. duodenalis* served as a strategic approach, as their probably shared transmission pathways make this group more likely to host *D. fragilis*. Since routine microscopy often fails to identify this protist, molecular analysis of these samples allowed us to quantify previously hidden coinfections and evaluate the true epidemiological impact of *D. fragilis*. Nonetheless, this approach entails key limitations of the present study: the absence of a non-parasitized control group, which precludes us from calculating the prevalence in the general symptomatic population or the prevalence of real coinfection. Consequently, our study population may represent a highly selected cohort rather than the general symptomatic population. Therefore, the reported 34.5% frequency of *D. fragilis* should be interpreted within the specific context of coinfections and should not be misconstrued as the baseline prevalence of this protist among unselected symptomatic subjects. Moreover, because all analyzed patients already harbored other protists, we are unable to establish a direct correlation between coinfection and clinical manifestations, or to safely attribute the development of symptoms solely to the presence of *D. fragilis*, making it impossible to determine if they were caused by *D. fragilis*, the coinfecting protist, or alternative causes.

We did not genetically characterize the *Blastocystis* sp. and *G. duodenalis* isolates, preventing us from investigating potential links between the molecular characterization of these two protists and coinfection. However, previous studies on *Blastocystis* sp. have not found such associations [60]. Regarding *D. fragilis*, a single genetic locus was studied for genotyping. Although this is currently the standard and most reliable method, it provides a highly conserved view of the genome and might mask intra-genotypic variation or mixed genotypic. Advanced approaches like Multilocus Sequence Typing (MLST) [7] will be crucial for obtaining more comprehensive genotypic information to search for a greater genetic diversity; however, available evidence indicates that alternative constitutive genes traditionally used in MLST, such as actin or EF-1α, lack utility in detecting these variations due to the very low sequence divergence between genotypes (approximately 3%) [13].

Finally, the possibility of cross-reactions in PCR assays with other trichomonads should not be overlooked. Although this phenomenon has been more frequently reported in isolates derived from animal sources, the difficulty in achieving sequencing-based confirmation in human samples yielding late Ct qPCR-positive results does not preclude the presence of such interferences [67].

The most comprehensive and recent review on the pathogenic role of *D. fragilis* highlights that the protist’s behavior is diverse and dependent on external factors, suggesting that future research should investigate the specific conditions (host factors and intestinal microbiota) under which it exhibits pathogenicity [68]. Coinfections can interact synergistically or antagonistically with each other and with the rest of the gut microbiota [63]. Consequently, a thorough evaluation of coinfections in patients with *D. fragilis* is crucial to unravel the intricate relationships with other protists and parasites. Regarding treatment, some studies demonstrate that cure rates are significantly higher when using paromomycin compared to metronidazole; this is particularly evident in cases of coinfection with *E. vermicularis*, which may favor *D. fragilis* persistence and hinder eradication efforts. In such cases, treating both parasites concurrently is necessary to achieve successful therapeutic outcomes [10, 29]. In this context, further exploration of *D. fragilis* coinfection with other parasites and protozoa is warranted to optimize therapeutic guidelines and achieve superior clinical outcomes.

## Data Availability

The sequences obtained in this study have been deposited in GenBank under accession numbers PX606388-PX606409.
